# A nuclear target sequence capture probe set for phylogeny reconstruction of the charismatic plant family Bignoniaceae

**DOI:** 10.3389/fgene.2022.1085692

**Published:** 2023-01-09

**Authors:** Luiz Henrique M. Fonseca, Mónica M. Carlsen, Paul V. A. Fine, Lúcia G. Lohmann

**Affiliations:** ^1^ Departamento de Botânica, Instituto de Biociências, Universidade de São Paulo, São Paulo, Brazil; ^2^ Systematic and Evolutionary Botany Laboratory, Department of Biology, Ghent University, Ghent, Belgium; ^3^ Missouri Botanical Garden, Saint Louis, MO, United States; ^4^ University and Jepson Herbaria, and Department of Integrative Biology, University of California, Berkeley, Berkeley, CA, United States

**Keywords:** bait kit, probe design, sequence capture, target enrichment, HybPiper, phylogenomics

## Abstract

The plant family Bignoniaceae is a conspicuous and charismatic element of the tropical flora. The family has a complex taxonomic history, with substantial changes in the classification of the group during the past two centuries. Recent re-classifications at the tribal and generic levels have been largely possible by the availability of molecular phylogenies reconstructed using Sanger sequencing data. However, our complete understanding of the systematics, evolution, and biogeography of the family remains incomplete, especially due to the low resolution and support of different portions of the Bignoniaceae phylogeny. To overcome these limitations and increase the amount of molecular data available for phylogeny reconstruction within this plant family, we developed a bait kit targeting 762 nuclear genes, including 329 genes selected specifically for the Bignoniaceae; 348 genes obtained from the Angiosperms353 with baits designed specifically for the family; and, 85 low-copy genes of known function. On average, 77.4% of the reads mapped to the targets, and 755 genes were obtained per species. After removing genes with putative paralogs, 677 loci were used for phylogenetic analyses. On-target genes were compared and combined in the Exon-Only dataset, and on-target + off-target regions were combined in the Supercontig dataset. We tested the performance of the bait kit at different taxonomic levels, from family to species-level, using 38 specimens of 36 different species of Bignoniaceae, representing: 1) six (out of eight) tribal level-clades (e.g., Bignonieae, Oroxyleae, Tabebuia Alliance, Paleotropical Clade, Tecomeae, and Jacarandeae), only Tourrettieae and Catalpeae were not sampled; 2) all 20 genera of Bignonieae; 3) seven (out of nine) species of *Dolichandra* (e.g., *D. chodatii*, *D. cynanchoides*, *D. dentata*, *D. hispida*, *D. quadrivalvis*, *D. uncata*, and *D. uniguis-cati*), only *D. steyermarkii* and *D. unguiculata* were not sampled; and 4) three individuals of *Dolichandra unguis-cati*. Our data reconstructed a well-supported phylogeny of the Bignoniaceae at different taxonomic scales, opening new perspectives for a comprehensive phylogenetic framework for the family as a whole.

## 1 Introduction

The plant family Bignoniaceae is a conspicuous and charismatic element of the tropical flora. The family has 85 genera and 850 species of shrubs, lianas, and trees (http://www.theplantlist.org) and a history of diversification that was broadly influenced by the colonization of different habitats and biogeographic regions (Lohmann et al., 2013; Olmstead, 2013; Thode et al., 2019; Francisco and Lohmann, 2020; Calió et al., 2022; Ragsac et al., 2022). The family is known for its conspicuous flowers, which are variable morphologically due to specialized plant-animal interactions associated with pollination (Gentry, 1990), and diverse fruit morphology associated with different dispersal systems (Zjhra et al., 2004; Farias-Singer, 2007; Ragsac et al., 2021). The great diversity and high homoplasy of Bignoniaceae’s reproductive morphology, the main characters used to circumscribe genera and supra-generic groupings within the family, led to considerable taxonomic confusion (reviewed by Gentry, 1980; Lohmann, 2006; Lohmann and Taylor, 2014). The availability of broad-scale phylogenies for the Bignoniaceae recently allowed for a re-circumscription of lineages in the family and the recognition of monophyletic tribes (Olmstead et al., 2009) and genera (Lohmann, 2006; Grose and Olmstead, 2007a).

To date, phylogenetic inference in this plant group has mainly relied on plastid markers (e.g., *mat*K, *ndh*F, *rbc*L, *rpl*32-*trn*L, *trn*L-F) or a few nuclear regions, such as the multi-copy nuclear ribosomal (ITS) or the low copy gene *Pep*C (e.g.; Lohmann, 2006; Olmstead et al., 2009; Fonseca and Lohmann, 2015; Francisco and Lohmann, 2020; Calió et al., 2022; Ragsac et al., 2021; Ragsac et al., 2022). Robust phylogenies were recovered for the family and infra-familial levels based on these markers, providing a framework to test tribal and generic limits within this plant clade. Traditionally recognized tribes such as Tecomeae emerged as paraphyletic (Spangler and Olmstead, 1999; Olmstead et al., 2009), while traditionally recognized genera were also shown to not represent monophyletic groupings, leading to extensive taxonomic changes, especially in the tribes Bignonieae (Lohmann, 2006; Lohmann and Taylor, 2014), and Tecomeae (Grose and Olmstead, 2007a; Grose and Olmstead, 2007b). However, the limited number of informative sites provided by the DNA regions traditionally used to reconstruct phylogenetic relationships within the Bignoniaceae prevents a thorough understanding of phylogenetic relationships within this family. Recalcitrant relationships and poorly supported branches are observed in deeper phylogenetic nodes or even within more recently diverging lineages (e.g., Lohmann, 2006; Olmstead et al., 2009), highlighting the need for a higher number of informative characters for phylogeny reconstruction at different taxonomic levels. While a few Bignoniaceae phylogenetic studies have incorporated plastome data (e.g., Fonseca and Lohmann, 2018; Thode et al., 2019; Fonseca and Lohmann, 2020), or thousands of nuclear loci (Dong et al., 2022) to reconstruct phylogenetic relationships within individual Bignoniaceae genera, no phylogenetic study to date has used genomic data to reconstruct a robust phylogeny of the family as a whole.

High throughput DNA sequencing technologies coupled with advances in bioinformatics are revolutionizing the field of evolutionary biology (Soltis et al., 2013) and nuclear genomes are increasingly available for non-model species (Fonseca and Lohmann, 2018; Anderman et al., 2020). Despite those advances, complete genomes are still not affordable, and the computational time necessary to analyze the massive amounts of genomic data remains prohibitive (McKain et al., 2018; Anderman et al., 2020). To circumvent these limitations, strategies of genome reduction that allow the incorporation of hundreds of thousands to millions of base pairs have been described. For example, multiplex PCR (Urive-Convers et al., 2016), RAD-seq (Davey and Blaxter, 2010; Eaton and Ree, 2013), RNA-seq (Wen et al., 2013), and target capture-based approaches (Faircloth et al., 2012; Weitemier et al., 2014) are allowing researchers to focus their sequencing efforts on loci that are useful to address different taxonomic, evolutionary, or biogeographical questions. These genome reduction approaches increase the cost-effectiveness of projects and improve phylogenetic resolution by allowing the inclusion of the most informative data and an increase in the number of taxa (Soltis et al., 2013; McKain et al., 2018; Anderman et al., 2020).

Target sequence capture approaches are a promising tool for studying evolutionary relationships in non-model organisms, enabling researchers to maximize the number of informative characters despite limited genomic resources (Anderman et al., 2020). This approach is cost-effective when compared to genome sequencing, allowing hundreds of pre-selected target loci to be obtained for dozens of specimens at once. Gene capture approaches have been quite successful in multiple plant phylogenetic studies, and customized bait kits are now available for those groups (Weitemier et al., 2014; Heyduk et al., 2016; Carlsen et al., 2018; Chau et al., 2018; Bagley et al., 2020; Jantzen et al., 2020; Christe et al., 2021; Eserman et al., 2021; Ogutcen et al., 2021; Yardeni et al., 2022). A universal bait kit designed to tackle conserved genes is also available for angiosperms as a whole (i.e., Angiosperms353; Johnson et al., 2019). The choice between clade-specific or universal bait kits depends on the questions being addressed and the degree of divergence among the studied taxa (Yardeni et al., 2022), with custom kits being more appropriate to resolve phylogenetic relationships at shallow scales (Eserman et al., 2021; Yardeni et al., 2022). The inclusion of universal kits in the design of custom probe sets (e.g., Jantzen et al., 2020; Baker et al., 2021; Christe et al., 2021; Eserman et al., 2021; Ogutcen et al., 2021), opens up the possibility of integrating data across flowering plants, while resolving phylogenetic relationships at different taxonomic scales.

Here, we developed and tested the first nuclear target sequence capture probe set for phylogenetic inference across the entire family Bignoniaceae. We obtained sequence data for 762 genes, representing 329 nuclear genes (1307 putative exons) selected following the Hyb-Seq protocol (Weitemier et al., 2014), 348 nuclear genes from the Angiospems353 bait set (Johnson et al., 2019), and 85 low-copy functional genes with implications for reproductive and vegetative organ development, and biochemical synthesis. Of the 762 genes selected, 677 are putatively single or low-copy nuclear genes. We tested the utility of the new bait set for phylogeny reconstruction at different taxonomic scales, including tribal, generic, and species levels. For the broadest taxonomic scale, we sampled species from six (out of eight) Bignoniaceae tribal-level clades (i.e., Bignonieae, Oroxyleae, Tabebuia Alliance, Paleotropical Clade, Tecomeae, and Jacarandeae), only Tourrettieae and Catalpeae were not sampled (Olmstead et al., 2009). We also sampled all 20 genera recognized in the tribe Bignonieae (Lohmann and Taylor, 2014; Fonseca and Lohmann, 2019). Furthermore, we selected the genus *Dolichandra* Cham. as a test case for resolving species-level relationships within the family and sampled seven (out of nine) species of *Dolichandra*, only *D. unguiculata* (Vell.) L.G. Lohmann and *D. steyermarkii* (Sandwith) L.G. Lohmann were not sampled (Fonseca and Lohmann, 2015). This study aimed to: 1) test the efficiency of the designed bait set to capture targeted regions throughout Bignoniaceae; and 2) test whether the analyses of the captured loci improve phylogenetic resolution at three different evolutionary scales: the family Bignoniaceae, the tribe Bignonieae, and the genus *Dolichandra*.

## 2 Materials and methods

### 2.1 Sampling

We used the genomic resources available for four species of Bignoniaceae to select low to single-copy genes. The transcriptomes of *Kigelia africana* (Lam.) Benth, *Mansoa alliacea* (Lam.) A.H. Gentry, and *Tabebuia umbellata* (Sond.) Sandwith were obtained from the 1 KP project (Matasci et al., 2014). The partial nuclear genome of *Handroanthus impetiginosus* (Mart. ex DC.) Mattos was also used (Silva-Junior et al., 2018; GenBank: NKXS01000000). To evaluate the bait set designed here, we sampled 38 species of Bignoniaceae. Plant materials used for this study were collected in the wild and dried in silica gel or collected from living collections available at the Universidade de São Paulo (São Paulo, Brazil). Because our sampling scheme aimed to evaluate the usefulness of our probe set across the Bignoniaceae at different taxonomic levels, we sampled species from five out of the eight recognized tribal-level clades (Olmstead et al., 2009), as follows: 1) Oroxyleae (1 sp.); 2) Tabebuia Alliance (6 spp.), 3) Tecomeae (2 spp.), 4) Jacarandeae (1 sp.), and 5) Bignonieae (28 spp.). All 20 genera of tribe Bignonieae currently recognized were sampled, including *Dolichandra* for which seven out of the nine known species (Fonseca and Lohmann, 2015) were included (accession information available in Supplementary Table S1).

### 2.2 Selection of target loci

Loci were selected targeting regions consisting of orthologous low-copy nuclear protein-coding genes and aiming to reconstruct relationships at three evolutionary scales: tribal, generic, and species levels. Genes were selected using the Hyb-Seq pipeline (Weitemier et al., 2014). The probe design was based on data from the draft genome of *H. impetiginosus* combined with the three transcriptomes. Contigs from the draft nuclear genome were matched against those sharing sequence similarities from the transcriptomes individually using the program BLAT (Kent, 2002). The similarity threshold used for *K. africana*, *M. alliacea*, and *T. umbellata* were 0.92, 0.9, and 0.95, respectively. The Hyb-Seq protocol was originally designed to use genomic and transcriptomic data from the same species and used an original threshold value of 0.99% of similarity. To overcome this limitation, we tested different threshold values from 0.99 to 0.85 (0.01 of difference between steps). The values selected were based on the number of loci selected at the end of the pipeline. Thousands of exons and genes were obtained from the three transcriptomes. To reduce the size of the dataset, we selected genes with less than 10 introns and less than 2040 bp (equivalent to 34 probes for 2× tiling). We only kept genes recovered in at least two different datasets/transcriptomes using CD-HIT-EST 4.5 (with–c 0.9) (Fu et al., 2012). This step also maximizes the chance that the selected loci are shared within different species of the family. A total of 329 low to single-copy genes were selected.

The 353 universal loci available for Angiosperms were included using the original alignments (available at: https://github.com/mossmatters/Angiosperms353) (Johnson et al., 2019). Sequences of all Bignoniaceae transcriptomes were retained in each of the 353 original alignments, while sequences from other plant families were discarded. For 338 loci, at least one sequence of Bignoniaceae was found. When more than one Bignoniaceae species was found for a specific gene, the longest sequence was retained. For the 15 genes without Bignoniaceae sequences, one sample from a closely related family in Lamiales was included. All genes were investigated to evaluate the number of copies using the genome of *H. impetiginosus* and BLAT (Kent, 2002). Originally, all genes were included with one reference, although only 348 were assembled. Using Bignoniaceae as a reference to design specific baits for the Angiosperms353 panel, we saved thousands of baits in our final set.

Functional genes were selected using *Arabidopsis thaliana* (L.) Heynh. or closer relatives of the Bignoniaceae as reference (mainly from *Solanum lycopersicum* L.) (Supplementary Material S2). First the number of gene copies was evaluated in BLAT (Kent, 2002) using the genome of *H. impetiginosus* as reference. Putative orthologous sequences of the focal genes were examined using BLAT (Kent, 2002) and the three transcriptomes available. The longest and most similar sequences (threshold of 0.85) available were selected for each reference. For three genes (ALCATRAZ, FRIGIDA, and INDEHISCENT) no Bignoniaceae sequences were recovered and the original *Arabidopsis thaliana* (L.) Heynh. or *Solanum lycopersicum* L. sequences were used (Supplementary Material S2). Initially, a total of 89 low to single-copy functional genes were selected; however, four genes failed during the assembly, and 85 genes were used in subsequent analyses.

To evaluate if different datasets (e.g., Hyb-Seq, Angiosperms353, and functional) shared loci, we used CD-HIT-EST 4.5 (with–c 0.9) (Fu et al., 2012). Two genes were shared between the Angiosperms353 and the functional datasets; only the references from the former were maintained. The gene set was sent to Arbor Biosciences and used as a basis to synthesize the 80 bp 2× tiled bait set. The Bignoniaceae bait set is available from Arbor Biosciences (Ann Arbor, Michigan, United States), and the gene sequences used to generate the bait set are provided on GitHub (https://github.com/luizhhziul/BigBait). We selected 771 putative low to single-copy nuclear loci for our probe set. Nine genes failed to be assembled, leading to a final number of 762 genes with at least one copy assembled.

### 2.3 Library preparation, sequencing, and gene assembly

Total genomic DNA was extracted from silica-dried or fresh leaf tissue using the Invisorb® Spin Plant Mini Kit (Invitek, Berlin, Germany). To isolate enough DNA for sequencing, multiple extractions were performed for some samples, pooled, and concentrated by vacuum centrifugation. Total DNA was quantified using Qubit BR assay. DNA quality was evaluated using NanoDrop 2000 (Thermo Fisher Scientific) and gel electrophoresis. Library preparation and sequence capture were performed by the QB3 Genomics facility (University of California, Berkeley) using their high-throughput workflow. All samples were prepared into standard-sized libraries using Kapa Biosystems library preparation kits (using Covaris sonicator to fragment gDNA) and custom Unique Dual Indexes. Samples were multiplexed, and the captured fragments obtained using the Bignoniaceae bait kit pooled and sequenced on an Illumina NovaSeq 6000 S4 with 150 bp paired-end reads.

Illumina reads were demultiplexed at the sequencing facility, and low-quality reads were trimmed using Trimmomatic 0.35 (Bolger et al., 2014) with the SLIDINGWINDOW:10:20 and MINLEN:40 parameters. HybPiper 1.3.1 (Johnson et al., 2016) was used to obtain the gene sequences using the “reads_first.py” Python script through the following steps: quality filtering; map reads to target the gene references provided using BWA 0.7.17 (Li and Durbin, 2009); *de novo* contig assembly using SPAdes 3.6.1 (Bankevich et al., 2012); and, return supercontigs (exons + introns/intergenic-regions) and exon-only sequences per gene using Exonerate 2.4.0 (Slater and Birney, 2005). We retrieved FASTA files of exon-only and supercontig sequences containing all sampled species using the “retrieve_sequences.py” script. Summary statistics were calculated using the “hybpiper_stats.py” script. Three genes were excluded due to their low representation (i.e., they appeared in less than 70% of the species sampled).

HybPiper flagged 202 genes that might contain paralogs. Paralog warnings produced by the HybPiper pipeline for samples with multiple long contigs were further investigated using the HybPiper scripts paraloginvestigator.py and paralogretriever.py (Johnson et al., 2016). To check if these sequences represented true paralogs or alleles, we produced alignments using both sequences retrieved for the 202 genes using MAFFT 7.450 (Katoh and Standley, 2013) and generated phylogenetic trees using FastTree (Price et al., 2009) with default parameters (Johnson et al., 2016). Of the 202 genes, 82 were flagged in more than three specimens or showed phylogenetic evidence of paralogy; these genes were excluded. The gene set used for downstream analyses contained 677 single copy loci. Two datasets were generated: 1) “Exon-Only”, with just exons; and 2) “Supercontig”, with exons, complete or partial introns, and complete or partial intergenic regions. The number of Parsimony Informative Sites (PIS) for each gene alignment and combined alignments were obtained using the R package “ips” (Heibl, unpublished data). Saturation was evaluated using gene alignments, the function “dist.dna” of the “ape” package (Paradis et al., 2004), and the molecular model K80. The genetic distance between species of *Dolichandra* was also evaluated using the function “dist.dna” and the K80 model.

### 2.4 Phylogenomic analyses

Phylogenomic inferences were performed using the subset of 677 genes. Each step detailed here was conducted for either the Exon-Only, or the Supercontig datasets. Each gene sequence was aligned using MAFFT 7.450 (Katoh and Standley, 2013) with an automatic selection of alignment strategy and a maximum of 1,000 iterations. Bases with more than 75% of the species as missing data were deleted. Statistical properties of each alignment were evaluated using R (R Core Team, 2020). Alignments were concatenated using AMAS (Borowiec, 2016) to generate a super-matrix with all data combined. Maximum likelihood analyses were implemented in IQ-TREE 1.6.1 (Nguyen et al., 2015). For individual genes, model selection was performed before tree search in IQ-TREE using the command–m MPF and greedy algorithm (Lanfear et al., 2012). For combined Exon-Only and Supercontig super-matrices, the option–m TESTMERGE was used to select the best partition scheme before tree search. Ultrafast bootstrap (UFBoot) replicates were inferred for all analyses with 1,000 reanalyzes and–bnni option, performing additional steps to further optimize UFBoot trees using the nearest neighbor interchange (NNI) algorithm.

Super-matrix approaches can be inconsistent due to discordance in gene trees. These discordances can result from multiple processes and are commonly attributed to either incomplete lineage sorting (ILS) and/or hybridization (Pamilo and Nei, 1988; Galtier and Daubin, 2008), or phylogenetic errors (Shen et al., 2021). To infer the species tree from the set of gene trees available, a coalescent approach was performed using ASTRAL-III 5.6.3 (Mirarab et al., 2014; Zhang et al., 2018). Low-support branches (i.e., >30%) were collapsed to improve accuracy (Zhang et al., 2018). Branch support in ASTRAL-III was calculated using Local Posterior Probabilities (LPP). Gene congruence was evaluated using the gene concordance factor (gCF; Minh et al., 2020) using IQ-TREE 1.6.1 (Nguyen et al., 2015).

## 3 Results

### 3.1 Target enrichment with bait hybridization

**FIGURE 1:**
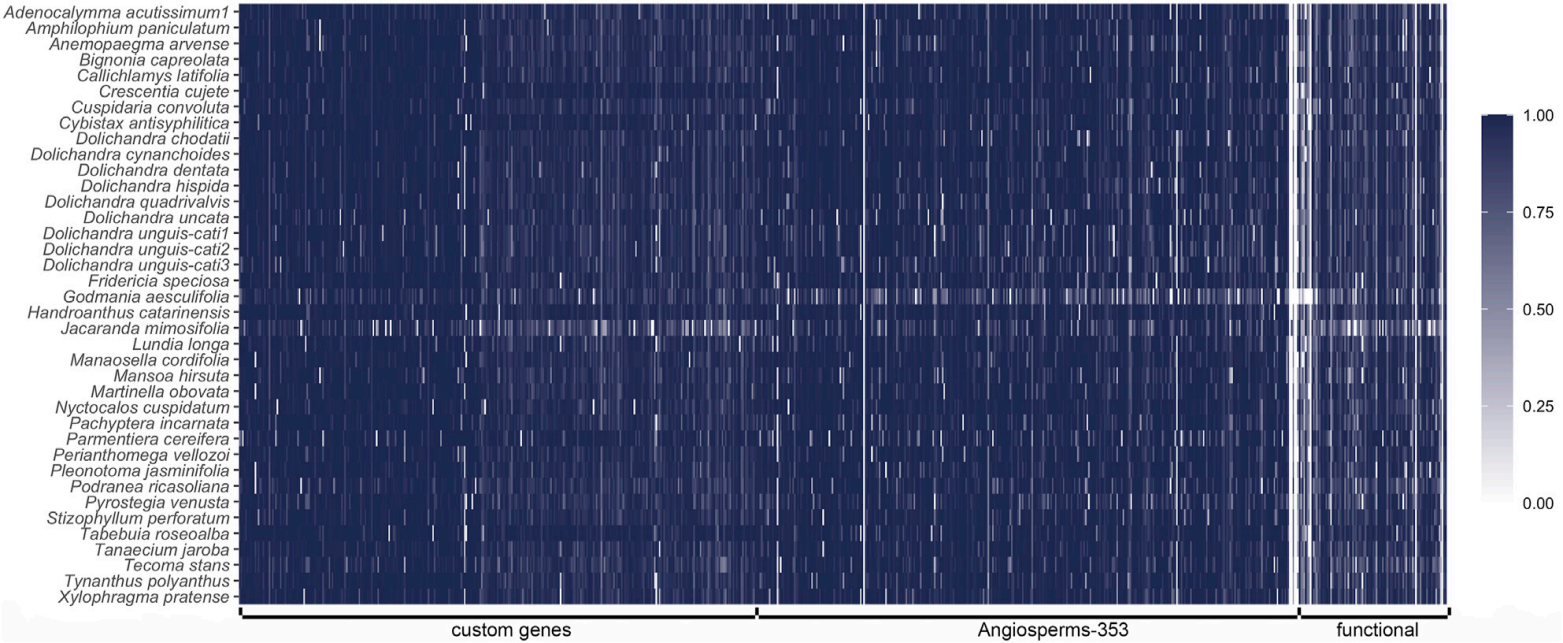
Recovered sequence heatmap for all the 771 genes targeted. Each row corresponds to a different specimen sampled, and each column correspond to a gene. Colors represent the length of the recovered sequence relative to the template sequence.

We recovered an average of 14,064,035.4 pair-end raw sequence data, with a maximum of 71,238,833 pair-end reads and a minimum of 3,077,426 pair-end reads. After the first quality filtering, we retained an average of 93.7% of the raw reads, with a maximum of 98.1% and a minimum of 76%. Raw reads for all accessions are available in GenBank Sequence Read Archive (SRA) under BioProject ID PRJNA909066. Baits showed high accuracy, and most raw reads mapped to a target gene (Table 1). An average of 77.4% of the reads mapped targets, with a maximum of 82.1% and a minimum of 4.4% (Table 1). Our bait set originally targeted 771 genes covered by 21,416 baits. Of the 771 genes originally targeted, 329 were specific for Bignoniaceae, 353 corresponded to genes in the Angiosperms353 bait kit, and 89 were known functional genes selected from the literature for this study (names of selected genes and references in Supplementary Material S2). We assembled 732–759 genes, with an average of 755 per specimen (Figure 1; Table 1). Nine genes failed for all species, including five genes from the Angiosperms353 set and four genes from the functional set. Recovery of the total reference gene length was 93.2%, with a maximum of 96% and a minimum of 82.7% (Table 1). A final array of 677 genes was used for gene alignments and phylogenetic tree searches after the removal of putative paralogs and genes not assembled in less than 70% of the species sampled.

**TABLE 1:** Summary statistics of sequencing success including the number of raw pair-end reads obtained, percentage of on-target reads, number of loci obtained, percentage of gene recovery, and number of loci retained after paralogs removed.

Tribe	Species	Pair-end reads	Percent of on-target reads	Number of loci obtained	Percent of recovery length	Number of loci retained
Bignonieae	*Adenocalymma acutissimum1*	18,401,073	76.1	757	93.3	676
Bignonieae	*Amphilophium paniculatum*	28,119,426	76.9	758	94.3	676
Bignonieae	*Anemopaegma arvense*	8,486,773	69.7	757	92.8	675
Bignonieae	*Bignonia capreolata*	4,863,524	80.1	759	94.5	676
Bignonieae	*Callichlamys latifolia*	11,089,886	75.2	757	94.7	676
Crescentieae	*Crescentia cujete*	6,770,767	80.4	759	96	676
Bignonieae	*Cuspidaria convoluta*	7,953,194	65.3	758	93.7	676
Tecomeae	*Cybistax antisyphilitica*	14,574,220	68.4	758	95.2	675
Bignonieae	*Dolichandra chodatii*	3,077,426	68.7	757	93.8	676
Bignonieae	*Dolichandra cynanchoides*	11,401,675	71.3	758	95	676
Bignonieae	*Dolichandra dentata*	9,614,141	79.4	758	94	676
Bignonieae	*Dolichandra hispida*	8,467,292	79.2	759	94	677
Bignonieae	*Dolichandra quadrivalvis*	10,465,488	80.7	758	93.8	676
Bignonieae	*Dolichandra uncata*	21,119,851	78.1	756	93	675
Bignonieae	*Dolichandra unguis-cati1*	15,336,452	80	755	92.1	674
Bignonieae	*Dolichandra unguis-cati2*	14,553,233	74.8	756	92.5	674
Bignonieae	*Dolichandra unguis-cati3*	15,517,980	49.1	757	92.5	675
Bignonieae	*Fridericia speciosa*	4,029,504	76.8	755	93.5	674
Tecomeae	*Godmania aesculifolia*	6,104,436	4.4	732	85.9	653
Tecomeae	*Handroanthus catarinensis*	13,546,481	66.6	758	94.9	675
Jacarandeae	*Jacaranda mimosifolia*	5,051,624	68.6	741	82.7	661
Bignonieae	*Lundia longa*	5,298,119	80.3	755	92.9	673
Bignonieae	*Manaosella cordifolia*	27,279,881	77.8	756	94.3	674
Bignonieae	*Mansoa hirsuta*	3,940,708	74.9	759	92.7	676
Bignonieae	*Martinella obovata*	5,605,255	75.6	758	94.6	676
Oroxyleae	*Nyctocalos cuspidatum*	55,042,136	79.6	754	94.1	673
Bignonieae	*Pachyptera incarnata*	7,932,766	75.6	756	94.1	674
Crescentieae	*Parmentiera cereifera*	71,238,833	80.8	754	93.1	673
Bignonieae	*Perianthomega vellozoi*	5,814,004	81.5	755	95	675
Bignonieae	*Pleonotoma jasminifolia*	7,456,086	78.3	756	94.2	675
Tecomeae	*Podranea ricasoliana*	16,729,542	78.7	755	91.9	675
Bignonieae	*Pyrostegia venusta*	5,081,154	66.8	757	91.7	675
Bignonieae	*Stizophyllum perforatum*	12,840,685	79.8	758	94.6	676
Tecomeae	*Tabebuia roseoalba*	9,587,299	82.1	756	95.6	675
Bignonieae	*Tanaecium jaroba*	23,112,074	76.1	758	94	676
Tecomeae	*Tecoma stans*	21,512,775	61.4	755	91.7	674
Bignonieae	*Tynanthus polyanthus*	11,207,016	80.2	757	93.7	675
Bignonieae	*Xylophragma pratense*	6,210,566	75	754	93.4	673

The mean length of the 677 genes was 192 bp to 6,316 bp long. Custom selected genes ranged from 940 bp to 2012 bp, while Angiosperms353 genes ranged from 192 bp to 4,324 bp, and functional genes ranged from 338 bp to 6,316 bp (Supplementary Figure S1). The Exon-Only alignment considering custom-selected genes, Angiosperm353 genes, and the functional genes was 875,075 bp long, of which 203,576 bp were parsimony informative at the family level, 121,268 bp at the tribal level, and 22,251 bp at the generic level (Table 2). The alignment considering the custom-selected genes exclusively was 348,264 bp long, of which 76,056 bp were parsimony informative at the family level, 64,790 bp at the tribal level, and 11,926 bp at the generic level (Table 2). The alignment considering the Angiosperms353 genes exclusively was 403,626 bp long, of which 94,933 bp were parsimony informative at the family level, 36,576 bp at the tribal level, and 6,740 bp at the generic level (Table 2). The alignment considering the functional genes exclusively was 123,185 bp long, of which 32,587 bp were parsimony informative at the family level, 19,902 bp at the tribal level, and 3,585 bp at the generic level (Table 2). Individual genes included 17 to 1,786 parsimony informative sites (Supplementary Figure S2). The pairwise genetic distance between species of *Dolichandra* ranged from 1% to 3.4%, while the pairwise genetic distance within *Dolichandra unguis-cati* L. ranged from 0.3% to 1% (Table 3).

**TABLE 2:** Number of aligned and parsimony informative bases for each dataset in each taxonomic scale.

	–	Informative sites		
	Size	Family	Tribe	*Dolichandra*
Angiosperms353	403,626	94,933	36,576	6,740
custom genes	348,264	76,056	64,790	11,926
functional	123,185	32,587	19,902	3,585
exon-only	875,075	203,576	121,268	22,251
Angiosperms353	1,084,724	462,527	283,030	42,457
custom genes	1,366,090	509,096	312,505	47,493
functional	361,143	143,370	92,873	14,880
supercontig	2,811,957	1,114,993	688,408	179,384

**TABLE 3:** Comparison of within-genus variation for all species of *Dolichandra*. Values below the diagonal are pairwise sequence divergences for the Supercontig dataset. Values above the diagonal are pairwise sequence divergences for the Exon-Only dataset. All values are in percentages.

	*D. chod*	*D. cyna*	*D. dent*	*D. hisp*	*D. quad*	*D. unca*	*D. ung.1*	*D. ung.2*	*D. ung.3*
*D. chodatii*	–	2.5	3.1	3.1	2.8	3.4	3.2	3.2	3.1
*D. cynanchoides*	3.8	–	2.6	2.6	2.2	2.9	2.7	2.7	2.6
*D. dentata*	4.8	4	–	1	2.7	2	1.6	1.6	1.5
*D. hispida*	4.8	4	1.5	–	2.7	2	1.6	1.6	1.6
*D. quadrivalvis*	4.3	3.5	4.1	4.1	–	3	2.8	2.8	2.7
*D. uncata*	5.3	4.5	3.1	3.1	4.6	–	2.1	2.1	2
*D. unguis-cati1*	4.9	4.1	2.4	2.5	4.2	3.2	–	0.3	1
*D. unguis-cati2*	4.9	4.1	2.3	2.4	4.2	3.1	0.4	–	1
*D. unguis-cati3*	4.9	4	2.3	2.4	4.2	3.1	1.6	1.6	–

### 3.2 Non-targeted sequences

Off-targeted regions from the “splash-zone” (i.e., protein-coding regions plus the complete or partial introns and intergenic regions) were 928 bp to 13,453 bp long. Custom selected genes ranged from 1,683 bp to 6,939 bp, while Angiosperms353 genes ranged from 928 bp to 10,816 bp, and functional genes ranged from 1,299 bp to 13,453 bp (Supplementary Figure S1). The Supercontig alignment was 2,811,957 bp long, of which 1,114,993 bp were parsimony informative at the family level, 688,408 bp at the tribal level, and 179,384 bp at the generic level (Table 2). The alignment containing the custom-selected genes exclusively was 1,366,090 bp long, of which 509,096 bp were parsimony informative at the family level, 312,505 bp at the tribal level, and 47,493 bp at the generic level (Table 2). The alignment with the Angiosperms353 genes exclusively was 1,084,724 bp, of which 462,527 bp were parsimony informative at the family level, 283,030 bp at the tribal level, and 42,457 bp at the generic level (Table 2). The alignment with the functional genes exclusively was 361,143 bp long, of which 143,370 bp were parsimony informative at the family level, 92,873 bp at the tribal level, and 14,880 bp at the generic level (Table 2). Individual genes included 160 to 5,544 parsimony informative sites (Supplementary Figure S2). The pairwise genetic distance between species of *Dolichandra* ranged from 1.5% to 5.3%, while the pairwise genetic distance within *D. unguis-cati* ranged from 0.4% to 1.6% (Table 3). Only 14 alignments were flagged with linear regression values below 0.7% using the molecular model K80 when Supercontig data was evaluated for saturation. Trees obtained for all 14 regions flagged were highly concordant with the concatenated tree and the species trees. As a result, the 14 genes were kept as part of the 677 set, allowing comparisons between the Exon-Only and Supercontig datasets due to the equivalent number of genes sampled.

### 3.3 Phylogenomic reconstructions

#### 3.3.1 Topologies inferred using the Exon-Only dataset

**FIGURE 2:**
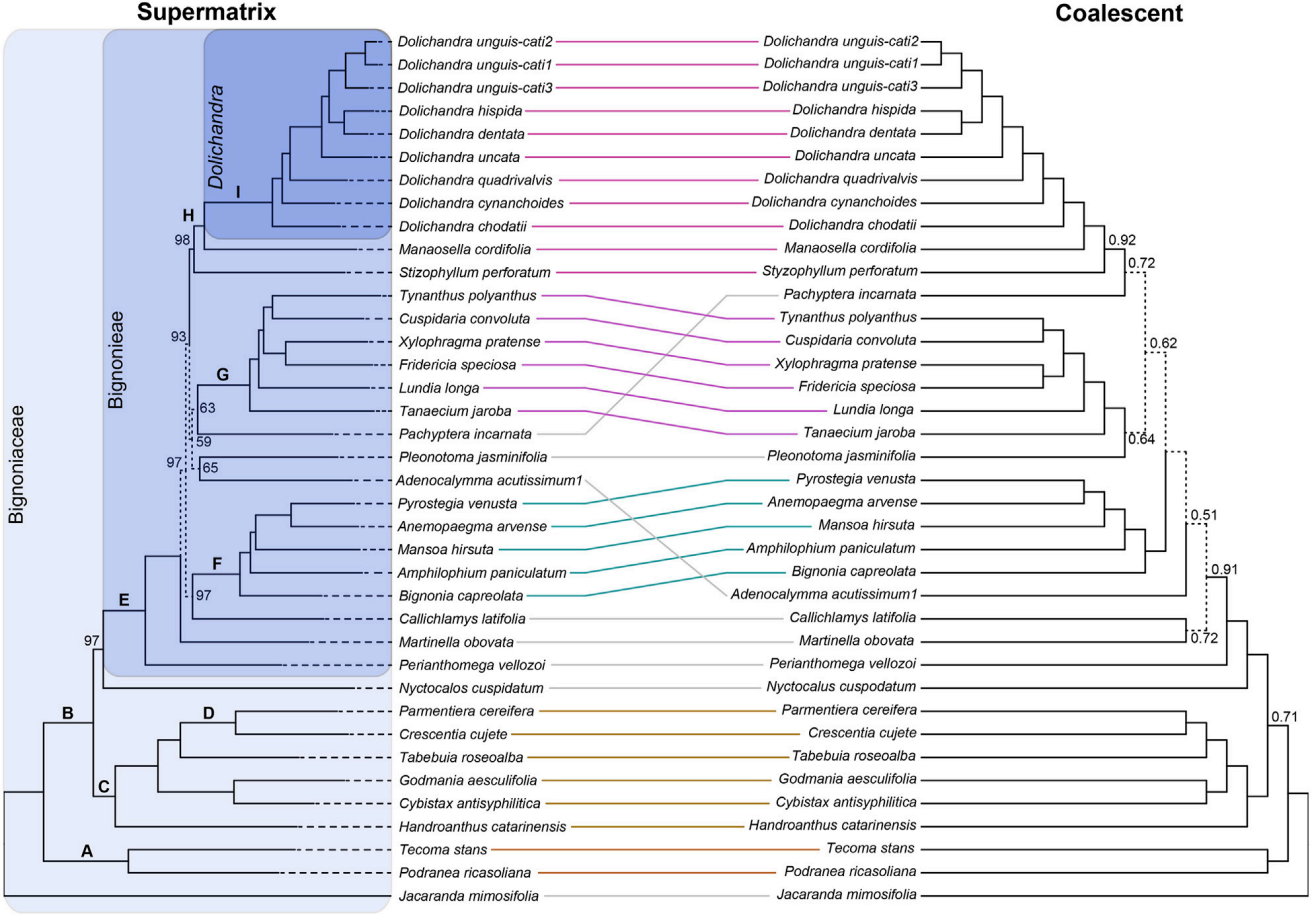
Tanglegram comparisons of phylogenies obtained using a supermatrix approach and IQ-TREE, and a coalescent approach and ASTRAL-III of the Exon-Only dataset. The supermatrix result includes ultra-fast bootstrap proportion (UFBoot) support values. The coalescent result is labeled with local posterior probabilities (LPP). Values above branches with maximum support of the UFBoot and LPP are not shown. Branches labeled on the supermatrix tree are discussed in the text. Shaded boxes enclose the entire Bignoniaceae family; the tribe Bignoniaeae; and the genus *Dolichandra*.

The single ML tree derived from the analysis of the concatenated Exon-Only dataset with 677 genes and 875,075 bp was well resolved, with most branches receiving high values of UFBoot (>90%). The me an UFBoot value for the entire tree is 96.4%. The tree was rooted with *Jacaranda mimosifolia* D. Don following previous phylogeny reconstructions of the Bignoniaceae (Olmstead et al., 2009). Tribe Bignonieae (Figure 2, clade E), tribe Crescentieae (Figure 2, clade D), tribe Tecomeae, and the entire Tabebuia Alliance clade emerged as monophyletic with maximum UFBoot support. All branches at the tribal level received maximum support, except for the clade composed of Bignonieae and Oroxyleae (*Nyctocalos cuspidatum* Miq.) with 97% of support. Most branches within tribe Bignonieae received maximum support from UFBoot. Exceptions were branches on the “backbone” of the tree or small clades with few genera, which are among the shortest branches of the tree. *Dolichandra* emerged as monophyletic with maximum support. All branches within the genus received maximum UFBoot support, including the relationships among the three terminals of *D. unguis-cati* (Figure 2).

The species trees obtained using a coalescent approach, and the supermatrix tree recovered similar topologies. The mean LPP value for the entire tree is 0.936. Differences in tree topology were usually poorly supported by both UFBoot and LPP, except for clade B (Figure 2), which showed maximum support in the concatenated tree, and 0.71 support in the coalescent tree. Phylogenetic relationships at the tribal-level, generic-level within clades F, G, and H, and species-level were concordant (Figure 2). All branches within *Dolichandra* received maximum support, including branches within *D. unguis-cati*. Gene tree congruence/conflict was evaluated visually and quantitatively for each node.

#### 3.3.2 Topologies inferred using the supercontig dataset

**FIGURE 3:**
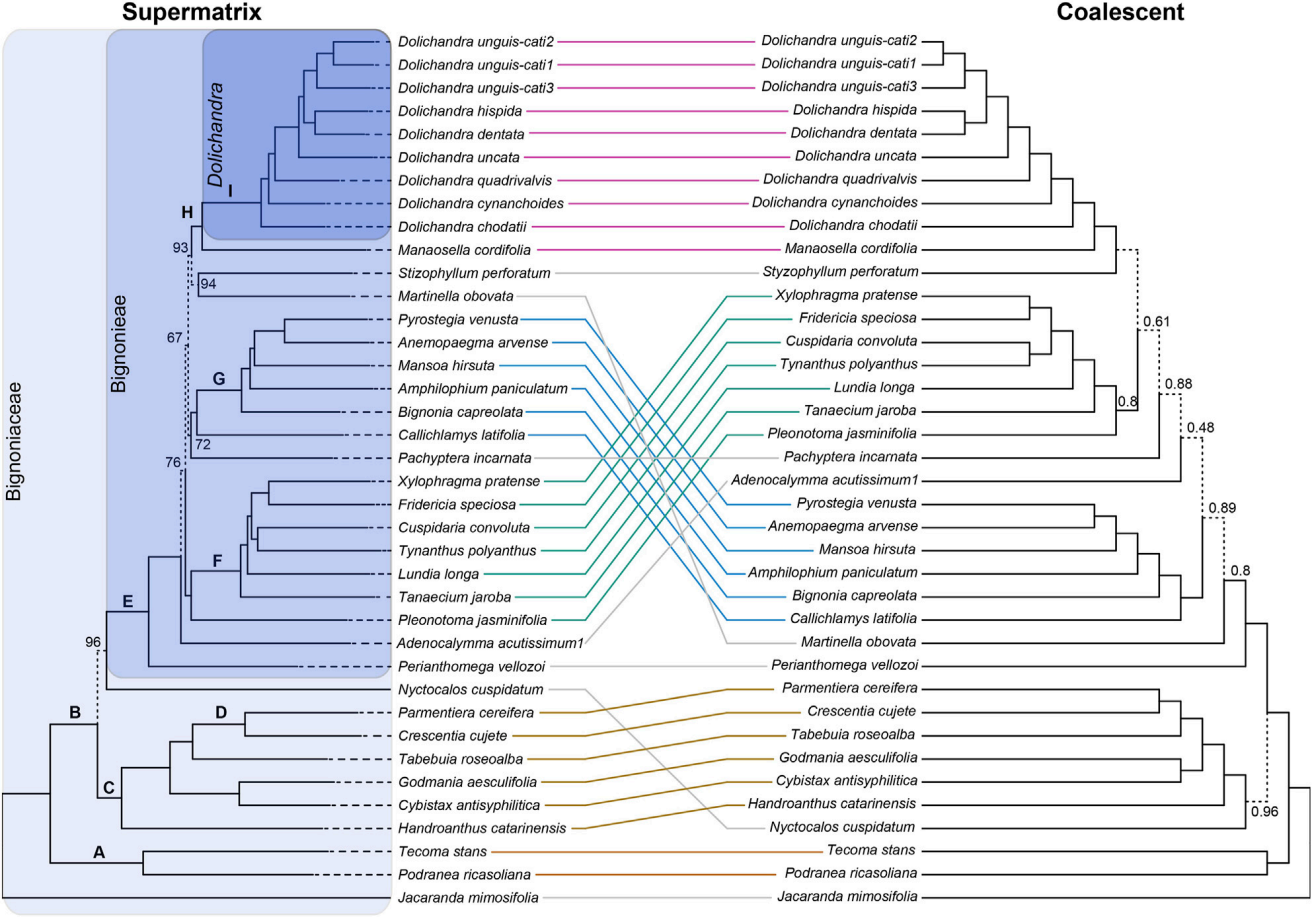
Tanglegram comparisons of phylogenies obtained using a supermatrix approach and IQ-TREE, and a coalescent approach and ASTRAL-III of the Supercontig dataset. The supermatrix result includes ultra-fast bootstrap proportion (UFBoot) support values. The coalescent result is labeled with local posterior probabilities (LPP). Values above branches with maximum support of the UFBoot and LPP are not shown. Branches labeled on the supermatrix tree are discussed in the text. Shaded boxes enclose the entire Bignoniaceae family; the tribe Bignoniaeae; and the genus *Dolichandra*.

The ML tree derived from the analysis of the concatenated Supercontig dataset with 677 genes and 2,811,957 bp is well resolved, with most branches supported by high UFBoot values (>90%). The mean UFBoot value for the entire tree is 97.2%. Strongly supported branches recovered the same relationships as the Exon-Only dataset. When tribal-level clades are considered, the clade composed of Bignonieae plus *N. cuspidatum* (Oroxyleae) is the only one with UFBoot of 96%. Most branches within tribe Bignonieae received maximum UFBoot support, except for nodes on the “backbone” of the tree, or small clades with few genera; these poorly supported branches are associated with the shortest branches of the tree. *Dolichandra* emerged as monophyletic with maximum support. All branches within the genus received maximum UFBoot support, including the phylogenetic relationship among the three terminals of *D. unguis-cati* (Figure 3).

The species tree derived from the coalescent approach and concatenated tree recovered a similar topology. The mean LPP value for the entire tree is 0.954. Differences in tree topology were usually poorly supported by both UFBoot and LPP. *Nyctocalos cuspidatum* now emerged as sister to clade C, being the only branch among tribal-level clades without maximum support of LPP (0.96). Most branches within tribe Bignonieae received maximum support of LPP. As recovered by the Exon-Only analyses analyses and the combined Supercontig analysis, the short branches of the “backbone” of Bignonieae are poorly supported. Clades F, G, and H were recovered with maximum support of LPP. Phylogenetic relationships within these clades also received maximum support of LPP, including the genus *Dolichandra* (Figure 3).

#### 3.3.3 Gene conflicts

**FIGURE 4:**
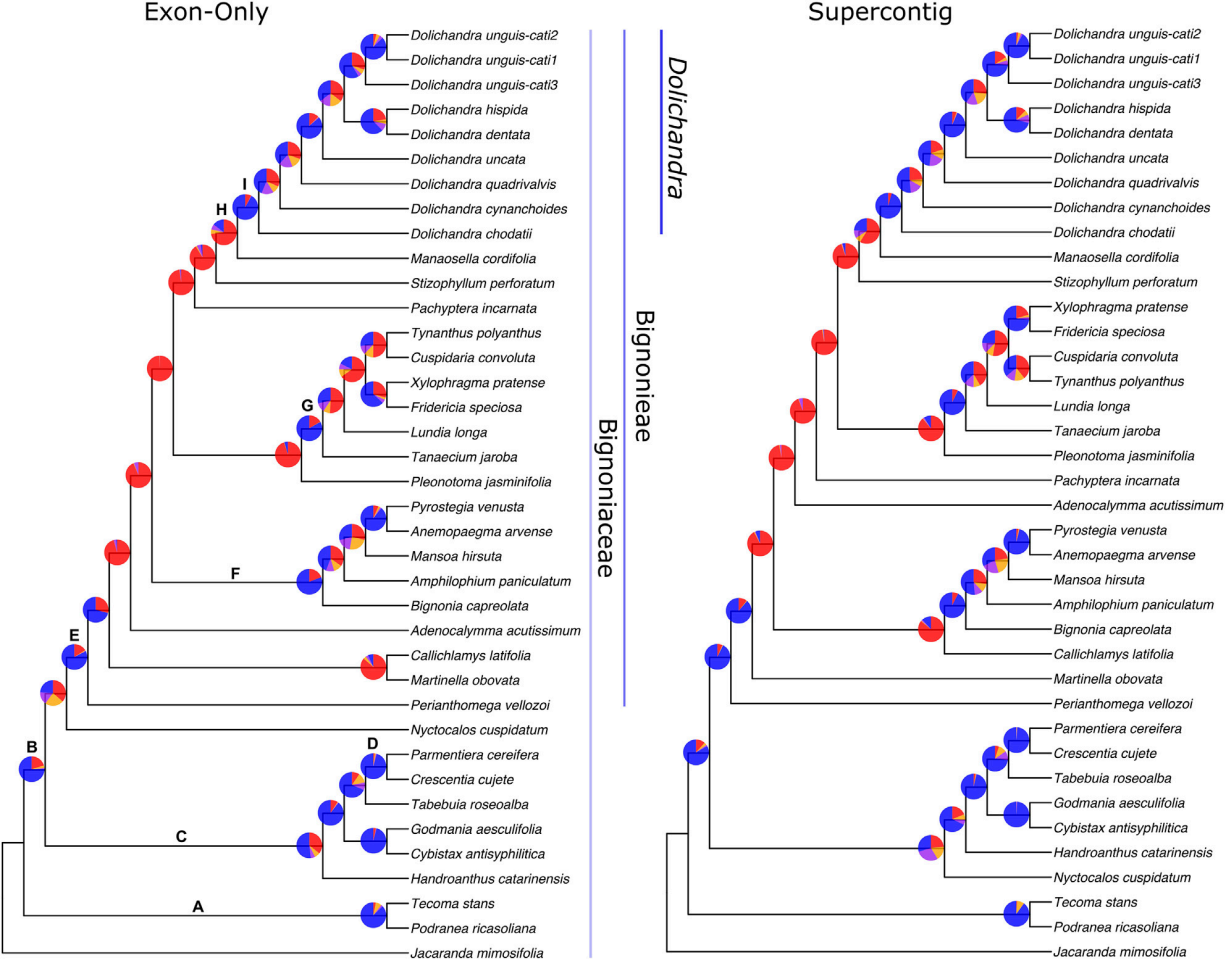
Phylogenies obtained through a coalescent approach and Exon-Only and Supercontig datasets. Gene concordance factor (gCF) values shown as pie charts. For gCF pie charts, blue represents the proportion of gene trees concordant with that branch, purple represents the proportion of gene trees concordant with the first alternative quartet, orange represents the proportion of gene trees concordant with the second alternative quartet, and red represents the gene discordance support due to polyphyly.

For the Exon-Only dataset, phylogenetic relationships among tribal-level clades were supported by more than 300 genes in all cases. The only exception is the Bignonieae + Oroxyleae clade, which was supported by 155 congruent genes. Conflicts were frequent on the “backbone” of Bignonieae, with six branches showing 15 or fewer genes that were congruent with the species tree. Clade H was supported with maximum values of UFBoot and LPP, however only 94 regions were congruent with this branch. Most of the genes were uninformative within this clade. Within *Dolichandra*, four branches were supported by more genes than the main alternative topology. In all cases, the species tree was congruent with at least 226 genes concordant with that branch (Figure 4).

For the Supercontig dataset, conflicts among genes were common in the “backbone” of Bignonieae, with eight clades supported by only 77 or less genes, with a higher number of genes supporting minor conflicts. Conflicts were also common within clade F, with three out of five clades having a higher number of genes supporting other resolutions of the quartet (Figure 4).

## 4 Discussion

Hybridization capture-based technologies are enabling the retrieval of hundreds of nuclear loci from diverse plant lineages (e.g., Soltis et al., 2013; McKain et al., 2018; Johnson et al., 2019; Anderman et al., 2020). Target enrichment approaches have been used to resolve phylogenetic relationships at many different levels, from universal bait kits for all flowering plants (Johnson et al., 2019), to custom kits designed for specific clades. Custom kits for targeted plant families such as Gesneriaceae (Ogutcen et al., 2021), Orchidaceae (Eserman et al., 2021), or Sapotaceae (Christe et al., 2021) and less comprehensive clades, such as the genus *Burmeistera* H. Karst. & Triana (Bagley et al., 2020), or *Dioscorea* L. (Soto Gomez et al., 2019) are available to date. The specificity of custom bait kits usually allows for a higher recovery rate in the targeted regions, with recovery values reaching up to 99.6% in *Dioscorea* (Soto Gomez et al., 2019). Here we provide the first bait kit for the tropical plant family Bignoniaceae, recovering up to 677 single-copy nuclear genes for phylogenetic analyses. The efficiency of our targeted sequence capture baits was extremely high, with a mean value of 98% of the genes recovered (Table 1). The kit included baits targeting three different gene sets. The first set is composed by 329 custom selected genes obtained using available genomic resources and the protocol described by Weitemier et al. (2014). The second set includes genes previously selected by the Angiosperms353 group (Johnson et al., 2019), with probes designed here specifically for Bignoniaceae. The last set is composed of low to single-copy functional genes. The bait kit was applied to 38 species of Bignoniaceae and aimed to resolve phylogenetic relationships from tribe to species-level. Most clades recovered on different levels of the tree received maximum support.

### 4.1 Capture efficiency

Our Bignoniaceae bait kit enabled the sequencing of 762 genes and up to 959,346 targeted base pairs (Table 1). The proportion of on-target reads, and the number of genes recovered for each specimen sampled can measure the efficiency of the capture reaction. Here, we obtained 73% (4.4–82.1) of on-target reads on average. This is a robust result compared to other angiosperms clades (e.g., 31.6% in *Dioscorea*, Soto Gomez et al., 2019; 48.6% in *Euphorbia*, Villaverde et al., 2018), revealing an efficient selection of targeted DNA fragments. The percentage of genes recovered is even higher, with a mean value of 98% and only two specimens obtaining less than 97.8% of the genes (Figure 1; Table 1). Nine genes (1.4% of total genes) failed to be assembled for all the species, including five genes from the Angiosperms353 set, and four genes from the functional set. Of the nine genes, eight used references outside Bignoniaceae due to a lack of sequences within the family. The average recovery of total length was of 93.2%. The species with the least data obtained was *J. mimosifolia*, with 82.5% of the reference recovered. *Godmania aesculifolia* (Kunth) Standl, the species with the fewest on-target reads (4.4%), recovered 85.9% of the reference size (Table 1). The result obtained here is excellent when compared to other studies (e.g., 78.6% in *Dioscorea*, Soto Gomez et al., 2019; 73% in *Euphorbia*, Villaverde et al., 2018). This result shows how the bait set is robust to capture a large number of genes, even when the enrichment reaction did not meet expectations (e.g., for *G. aesculifolia* with 4.4% of on-target reads). Here we applied a great depth of sequence, higher than that applied in other studies (e.g., Soto Gomez et al., 2019; Jantzen et al., 2020; Sanderson et al., 2020; Eserman et al., 2021). The sequencing depth and laboratory protocols applied during library preparation and enrichment reaction are relevant variables controlling the number of reads on target, the number of genes recovered, or the total length of the genes compared to the reference (Johnson et al., 2016; Anderman et al., 2020). Comparisons between studies are limited because of the many variables involved during wet lab steps and sequencing; however, our results illustrate that we have designed an extremely efficient bait kit for molecular phylogenetic studies of Bignoniaceae.

### 4.2 The paralogs

The number of genes used for phylogenetic analyses was reduced to 677 after removing paralogs and genes with sequences present in less than 70% of the species (Table 1). HybPiper flagged 202 genes that represented putative paralogs, a number that is similar to that recovered by other studies (135 of 681 genes in *Burmeistera*, Bagley et al., 2020; 219 of 830 genes in Gesneriaceae, Ogutcen et al., 2021). These genes were evaluated using phylogenetic trees. We found little evidence of paralogy, with most trees showing sequences from the same sample grouped together. Of the 202 genes, 82 were removed due to paralogy or because they showed more than two specimens flagged as paralogous. Many putative alleles were obtained, which can be explained by the great gene coverage obtained for all species (Johnson et al., 2016; Table 1).

The decision to exclude genes with paralogs can be considered conservative, removing a substantial amount of sequence data that could be used in phylogenetic inferences. Strategies to select and incorporate paralogs are available (Yang and Smith, 2014; Moore et al., 2018; Karimi et al., 2020; Morales-Briones et al., 2021), allowing the expansion of the final gene set using gene tree-guided orthology identification. Criteria such as “Monophyletic outgroups” or “Rooted ingroups” could be used to identify paralogs (Yang and Smith, 2014; Morales-Briones et al., 2021). These different gene sequences could be used as paralog-specific references in HybPiper, recursively recovering orthologous sequences (e.g., Johnson et al., 2016; Karimi et al., 2020). A pilot study using this strategy was applied to the Bignoniaceae data generated here, adding 71 genes after two rounds of iteratively selecting orthologous and paralogous sequences. Although these data were not used in this paper for phylogenetic analyses, it highlights the potential of the genes flagged as paralogous by HybPiper for future phylogenetic studies within the family.

### 4.3 Bignoniaceae phylogenomics

We inferred phylogenomic relationships for all samples using two datasets: the “Exon-Only”, containing the targeted regions, and the “Supercontig”, containing the targeted regions + non-targeted regions composed of introns or intergenic regions. We also applied two methods to infer trees: a concatenation method using a supermatrix and a coalescent-based species tree estimation. Gene tree incongruence due to incomplete lineage sorting is a common pattern not accounted for by concatenation methods, which could result in high support for an incorrect topology (Degnan and Rosenberg, 2009). Coalescent approaches minimized this problem, representing a fundamental tool in phylogenomic studies using nuclear data (Karimi et al., 2020; Morales-Briones et al., 2021).

The trees obtained using both datasets and methods were generally very similar to each other (Figures 2, 3) and resembled previous phylogenetic findings. The phylogenetic relationships at the tribal-level were evaluated using representatives of five different tribes or clades. *Jacaranda mimosifolia* (tribe Jacarandeae) was used to root the tree. Tribes Bignonieae, Crescentieae, Tecomeae, and the Tabebuia Alliance clade emerged as monophyletic in all results with maximum support of UFBoot or LPP (Figures 2, 3). These findings corroborate previous results using Sanger-generated data (Lohmann, 2006; Olmstead et al., 2009; Ragsac et al., 2021). Tribes Bignonieae and Tecomeae have a consistent taxonomic history and are recognized by morphological synapomorphies (Gentry, 1976; Lohmann and Taylor, 2014; Ragsac et al., 2021). Tribe Tecomeae and the Tabebuia Alliance clade emerged as monophyletic here, corroborating the most recent phylogeny of the family (Olmstead et al., 2009). *Nyctocalos cuspidatum* (tribe Oroxyleae) emerged as sister of Bignonieae in most trees; however, the UFBoot support was not maximum for both datasets and the species tree obtained using the Supercontig dataset recovered *N. cuspidatum* as sister to clade C. An expanded sampling of Oroxyleae could help place this tribe within the Bignoniaceae with higher certainty (Olmstead et al., 2009). Other phylogenetic relationships at the tribal-level received maximum support and were congruent throughout the analyses (Figures 2, 3) suggesting a robust set of genes for phylogenetic studies at this level.

The robustness of the kit to resolve phylogenetic relationships at tribal-level clades of Bignoniaceae is also clear when the numbers of parsimony informative bases are considered. The Exon-Only dataset had 203,576 bp (23.2%) of informative sites at this level, while the Supercontig dataset reached 1,114,993 bp (39.6%). When custom selected and the Angiosperms353 genes are compared in terms of Exon-Only, the proportion of informative sites was 21.8% and 23.5%, respectively. For Supercontig, the proportions are 37.2% and 42.6% (Table 2). The proportions of phylogenetically informative sites (PIS) between custom selected and the Angiosperms353 datasets are similar, with a slight advantage for the Angiosperms353. This finding resonates previous results that showed that bait kits could be as informative as custom baits at comprehensive levels of the tree (Yardeni et al., 2022).

We sampled all 20 genera of tribe Bignonieae to evaluate the performance of the gene set at this level. Considering the results derived from the analyses of the Exon-Only and Supercontig datasets, the trees were robust, congruent with hundreds of genes (Figure 4), and showed branches that mostly received maximum support for both UFBoot and LPP (Figures 2, 3). The topology largely resembles the most comprehensive phylogenetic result for Bignonieae (Lohmann, 2006). Among the similarities are the sister relationship between *Perianthomega vellozoi* Bureau and the rest of the tribe. The clades “Multiples of Four” and “*Fridericia* and Allies” also emerged as monophyletic (Lohmann, 2006; Lohmann and Taylor, 2014); these phylogenetic relationships received maximum support for both metrics (Figures 2, 3). Other phylogenetic relationships were revealed for the first time by the new data, such as the clade that included *Dolichandra* as sister to *Manaosella cordifolia* (DC.) A.H. Gentry. Both genera are composed of lianas and conspicuous flowers with membranaceous calyces and infundibular corollas (Fonseca and Lohmann, 2015). Poorly supported clades recovered in previous studies (Lohmann, 2006) are also poorly supported here. Furthermore, new phylogenetic relationships were recovered in the “backbone” of the tree, but these relationships were poorly supported (Figures 2, 3). Incomplete lineage sorting appears to be a reasonable explanation for these recalcitrant regions of the tree (Suh et al., 2015; Moore et al., 2018); however, the results from ASTRAL-III were poorly supported for this region of the tree and revealed significant underlying genomic conflicts (Figures 2, 3, 4) suggesting that other processes might be shaping the tree.

At the generic-level, the bait kit resolved most phylogenetic relationships with maximum support, although some poorly supported short branches revealed significant conflicts between gene trees (Figure 4). This result highlights how diversification over short periods of evolutionary time may impact phylogeny reconstruction, despite the abundant molecular data available (Table 2). The Exon-Only dataset had 121,268 bp (13.8%) informative sites at the genus-level, and the Supercontig dataset had 688,408 bp (24.5%). For the Exon-Only dataset, the proportions of the custom-selected and Angiosperms353 genes were 18.6% and 9%, respectively. For the Supercontigs, the proportions were 22.9% and 26%, respectively. Overall, our findings indicate that the target genes of the Angiosperms353 bait kit are less variable at the genus-level; however, when non-targeted data are included, this bait kit has genes with higher PIS. The same trend is observed in *Dolichandra*, where the Exon-Only recovered 3.4% and 1.7% of PIS for the custom-selected and Angiosperms353 datasets, while the Supercontig recovered 3.4% and 3.9%, respectively (Table 2). These results are in line with earlier findings in *Buddleja* L. (Chau et al., 2018), *Burmeistera* (Balgley et al., 2020), *Cyperus* L. (Larridon et al., 2020), and Orchidaceae (Yardeni et al., 2022), where the universal bait kit worked as well as the custom kit or even better at the generic and infra-generic levels.

Within *Dolichandra*, all analyses recovered congruent results that were fully supported by UFBoot and LPP (Figures 2, 3). These findings corroborate earlier phylogenetic hypotheses obtained using plastid data (i.e., *ndh*F and *rpL*32-*trn*L). Interestingly, the topology of *Dolichandra* previously recovered based on a nuclear marker (i.e., *pep*C; Fonseca and Lohmann, 2015) is not fully concordant with the topology obtained here. The number of informative sites just for *Dolichandra* reached 22,251 bp for the Exon-Only data and 179,384 bp for the Supercontig dataset (Table 2). To evaluate the potential of the 677 genes used in this study for shallow taxonomic scales, we also evaluated within-genus pairwise distances. All within-genus comparisons showed values that were greater than 1% of sequence divergence. Even within species, 0.3% and 1% of sequence divergence was recovered for the Exon-Only, while 0.4% and 1.6% of sequence divergence was recovered for the Supercontig dataset. For these analyses, three specimens of *D. unguis-cati* from different localities were used (Supplementary Table S1). These findings highlight the potential utility of the bait kit for species delimitation and population studies, which is consistent with earlier findings based on the Angiosperms353 kit (Slimp et al., 2021).

### 4.4 To Bignoniaceae and beyond

The datasets “Exon-Only” and “Supercontig” recovered similar trees in all phylogenetic strategies (Figures 2, 3, 4), showing the presence of phylogenetic signal in both protein coding and the “splash zone” regions. The absence of saturated markers also reveals the utility of protein coding and non-coding regions at different levels, reaching the family level of the tree. Gains in UFBoot (96.4 *vs*. 97.2) and LPP (0.936 *vs*. 0.954) were marginal when both “Exon-Only” and “Supercontig” datasets were compared. *Dolichandra* was also recovered as monophyletic with all branches receiving maximum support in all scenarios. These results could suggest redundancy between datasets, however the addition of the thousands of base pairs from the “splash zone” will certainly be relevant to resolve phylogenetic relationships at shallow levels of the tree (Bagley et al., 2020; Ogutcen et al., 2021).

The use of the bait kit beyond Bignoniaceae is speculative, with possible applications inside the order Lamiales (Ogutcen et al., 2021) as suggested by the 12 of the 18 genes without Bignoniaceae references successfully assembled. The steps used here to select low copy genes are certainly reproducible in other plant groups. The Hyb-Seq protocol is wide established, requiring few genomic resources to select single/low copy genes (Weitemier et al., 2014). New pipelines could also be used to complement the gene pool selected by Hyb-Seq or as alternatives, such as MarkerMiner (Chamala et al., 2015), or AllMarkers (Kadlec et al., 2017). The strategy used to select family/clade specific genes from the 353 universal loci pool is also reproducible, with more than a thousand of transcriptomes covering the majority of angiosperm diversity available for gene selection (Johnson et al., 2019).

## 5 Conclusion

Here we provide the first bait kit designed to capture low to single-copy nuclear genes for the plant family Bignoniaceae. The kit incorporates novel markers designed specifically for this plant family using the Hyb-Seq protocol; the gene set of the Angiosperms353, with baits designed specifically for Bignoniaceae; and functional genes with regulatory roles at different stages of flower, fruit, and leaf development, as well as roles in biochemical synthesis (Supplementary Material S2). Our bait kit enables the capture of 762 genes, among which 329 are specific for Bignoniaceae, 348 are from the Angiosperms353 bait kit designed by Johnson et al. (2019), and 85 correspond to functional genes. We tested the effectiveness of the enrichment steps using 38 samples of Bignoniaceae from 36 different species. These taxa spanned five different tribes, 20 different genera within the Bignonieae, and seven species of the genus *Dolichandra*. Gene recovery was exceptionally high, enabling near complete on-target data in all different levels evaluated.

The approach implemented here validated the bait kit from tribal to species-level, recovering informative regions and robust phylogenetic relationships through different time scales. Resolving phylogenetic relationships with highly supported branches is a prerequisite for many downstream applications such as diversification, biogeographic, and evolutionary studies. Phylogenomic results could also update classifications and contribute to taxonomic studies. The Bignoniaceae-specific kit will be implemented in phylogenetic studies at species-level within the family. We aim to clarify the evolutionary history of morphological traits, biogeographic history, timing of origin, and many other open questions to be addressed in the family. The kit will also allow for data reuse and will contribute to ongoing efforts to assemble the plant tree of life using the Angiosperms353 kit (Baker et al., 2021). The kit developed here will also allow evo-devo and physiological studies, especially through the use of the set of 85 functional genes selected. Indeed, the newly developed probe set will allow many evolutionary questions to be addressed within the Bignoniaceae using a reliable phylogenomic framework.

## Data Availability

Sequence alignments and phylogenetic trees presented in this study can be found on Github: https://github.com/luizhhziul. Raw reads for all accessions are available in GenBank Sequence Read Archive (SRA) under BioProject ID PRJNA909066.
