# Supplementary material for: A nuclear target sequence capture probe set for phylogeny reconstruction of the charismatic plant family Bignoniaceae
Source: Front Genet. 2023 Jan 9;13:1085692. doi: 10.3389/fgene.2022.1085692 (PMC9869424; doi:10.3389/fgene.2022.1085692)
Supplement: Supplementary file 1 [file DataSheet1.PDF]

Table S1. Voucher information for all species sampled.

Species	Authority	Voucher
Adenocalymma acutissimum1	(Cham.) Miers	Fonseca, L. 208 (SPF)
Amphilophium paniculatum	(L). Kunth	Fonseca, L. 416 (SPF)
Anemopaegma arvense	(Vell.) Stellfeld ex J.F. Souza	Firetti, F. 241 (SPF)
Bignonia capreolata	L.	Lohmann, L. 356 (MO)
Callichlamys latifolia	(Rich.) K. Schum.	Kataoka, E. 8 (SPF)
Crescentia cujete	L.	Fonseca, L. 492 (SPF)
Cuspidaria convoluta	(Vell.) A.H. Gentry	Lohmann, L. 713 (MO)
Cybistax antisyphilitica	(Mart.) Mart.	Linhares, K. s.n. (SPF)
Dolichandra chodatii	(Hassl.) L.G. Lohmann	Fonseca, L. 136a (SPF)
Dolichandra cynanchoides	Cham.	Pace, M. 490 (SPF)
Dolichandra dentata	(K. Schum.) L.G. Lohmann	Fonseca, L. 124 (SPF)
Dolichandra hispida	(DC.) L.H. Fonseca & L.G. Lohmann	Fonseca, L. 27 (SPF)
Dolichandra quadrivalvis	(Jacq.) L.G. Lohmann	Fonseca, L. 273 (SPF)
Dolichandra uncata	(Andrews) L.G. Lohmann	Zardini, E. 36351 (MO)
Dolichandra unguis-cati1	L.	Fonseca, L. 164 (SPF)
Dolichandra unguis-cati2	L.	Fonseca, L. 135 (SPF)
Dolichandra unguis-cati3	L.	Fonseca, L. 104 (SPF)
Fridericia speciosa	Mart.	Zuntini, A. 311 (SPF)
Godmania aesculifolia	(Kunth) Standl.	Rodrigues, L. 699 (SPF)
Handroanthus catarinensis	(A.H. Gentry) S.O. Grose	Heiden, G. 1446 (SPF)
Jacaranda mimosifolia	D. Don.	Fonseca, L. 490 (SPF)
Lundia longa	(Vell.) DC.	Zuntini, A. 199 (SPF)
Manaosella cordifolia	(DC.) A.H. Gentry	Fonseca, L. 421 (SPF)
Mansoa hirsuta	DC.	Lohmann, L. 364 (SPF)
Martinella obovata	(Kunth) Bureau & K. Schum.	Kataoka, E. 310 (SPF)
Nyctocalos cuspidatum	Miq.	Young, K. 145 (SPF)
Pachyptera incarnata	(Aubl.) J.N.C. Franc. & L.G. Lohmann	Beyer, M. 293 (SPF)
Parmentiera cereifera	Seem.	Colli-Silva, M. 13 (SPF)
Perianthomega vellozoi	Bureau	Pace, M. 28 (SPF)
Pleonotoma jasminifolia	(Kunth) Miers	Gomes, B. 600 (SPF)
Podranea ricasoliana	(Tanfani) Sprague	Fonseca, L. 488 (SPF)
Pyrostegia venusta	(Ker Gawl.) Miers	Lohmann, L. 718 (MO)
Stizophyllum perforatum	(Cham.) Miers	Fonseca, L. 105 (SPF)
Tabebuia roseoalba	(Ridl.) Sandwith	Fonseca, L. 489 (SPF)
Tanaecium jaroba	Sw.	Nunes, A. 288 (SPF)
Tecoma stans	(L.) Juss. ex Kunth	Pace, M. 421 (SPF)
Tynanthus polyanthus	(Bureau) Sandwith	Medeiros, M. 40 (SPF)
Xylophragma pratense	(Bureau & K. Schum.) Sprague	Fonseca, L. 238 (SPF)

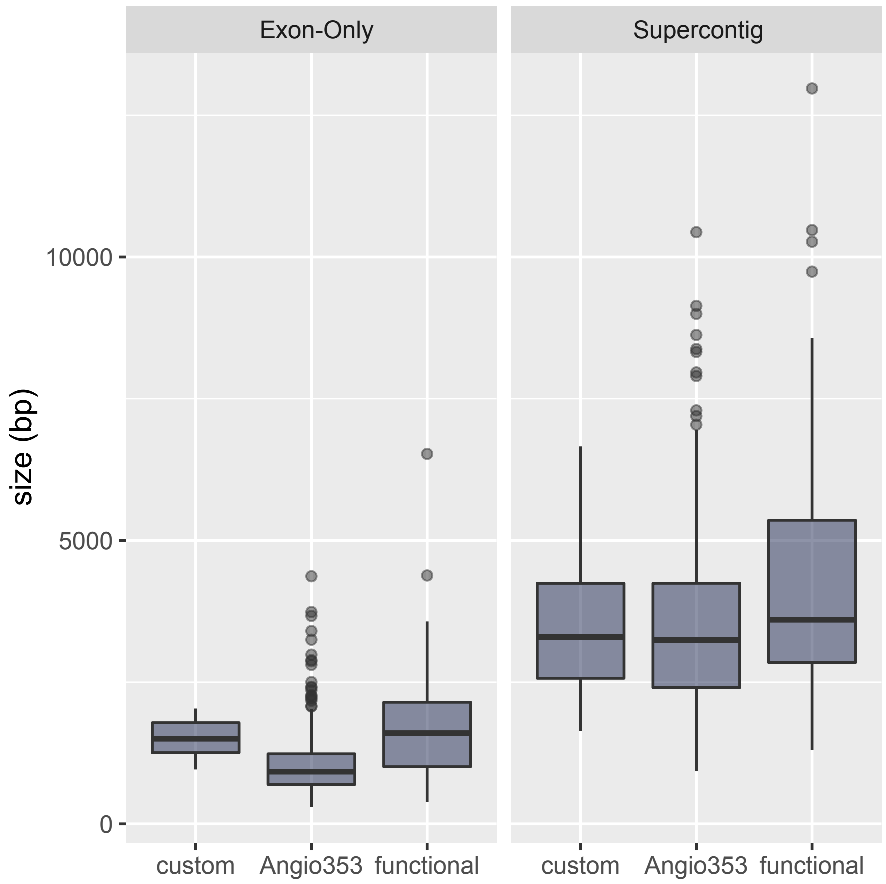

Figure S1 Size of the sequences obtained for each dataset considering all the specimens sampled. Custom selected, Angiosperms353, and functional genes are compared within each dataset.

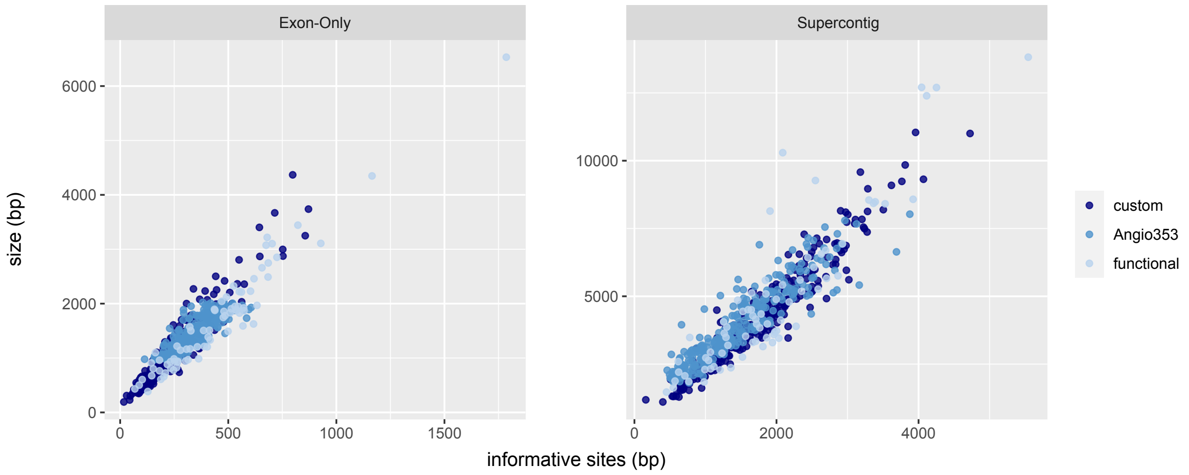

Figure S2 Size and number of parsimony informative sites for each of the 677 genes used for phylogenetic analyses. In dark blue the selected genes using the Hyb-Seq protocol; in steel blue the genes used in the Angiosperms353 panel; and in light blue the functional genes.
